# Targeting microRNAs with thymoquinone: a new approach for cancer therapy

**DOI:** 10.1186/s11658-021-00286-5

**Published:** 2021-10-09

**Authors:** Mina Homayoonfal, Zatollah Asemi, Bahman Yousefi

**Affiliations:** 1 Research Center for Biochemistry and Nutrition in Metabolic Diseases, Institute for Basic Sciences, Kashan University of Medical Sciences, Kashan, Islamic Republic of Iran; 2 Molecular Medicine Research Center, Tabriz University of Medical Sciences, Tabriz, Iran; 3 Department of Biochemistry, Faculty of Medicine, Tabriz University of Medical Sciences, Tabriz, Iran

**Keywords:** Thymoquinone, miRNA, Signaling pathway, Metastasis, Angiogenesis, Apoptosis, Epigenetic

## Abstract

Cancer is a global disease involving transformation of normal cells into tumor types via numerous mechanisms, with mortality among all generations, in spite of the breakthroughs in chemotherapy, radiotherapy and/or surgery for cancer treatment. Since one in six deaths is due to cancer, it is one of the overriding priorities of world health. Recently, bioactive natural compounds have been widely recognized due to their therapeutic effects for treatment of various chronic disorders, notably cancer. Thymoquinone (TQ), the most valuable constituent of black cumin seeds, has shown anti-cancer characteristics in a wide range of animal models. The revolutionary findings have revealed TQ’s ability to regulate microRNA (miRNA) expression, offering a promising approach for cancer therapy. MiRNAs are small noncoding RNAs that modulate gene expression by means of variation in features of mRNA. MiRNAs manage several biological processes including gene expression and cellular signaling pathways. Accordingly, miRNAs can be considered as hallmarks for cancer diagnosis, prognosis and therapy. The purpose of this study was to review the various molecular mechanisms by which TQ exerts its potential as an anti-cancer agent through modulating miRNAs.

## Introduction

According to the statistics of the Global Cancer Observatory, provided by the International Agency for Research on Cancer, the worldwide incidence and mortality rates of cancer in the year 2020 were estimated at almost 19.3 million and 10.0 million respectively [1]. Cancer, the second leading cause of death following cardiovascular diseases, is a fundamental health concern occurring as a consequence of converting normal cells into tumor ones. This transformation arises from numerous phases which result in pre-cancerous cell changes into a malignant status in certain parts of the human body [2]. The underlying mechanisms that may lead to occurrence and evolution of various kinds of cancers are entirely different and for the most part have not been thoroughly comprehended. Nevertheless, alterations in genetic and epigenetic regulatory pathways have been perceived as possible reasons for occurrence of numerous cancer types [3]. Correspondingly, various practical approaches including surgical, chemical drugs, radiation, immunological, non-coding RNAs targeting, and hormone therapies have been applied for cancer treatment based on the type and the development of different cancers [4].

**Fig. 1:**
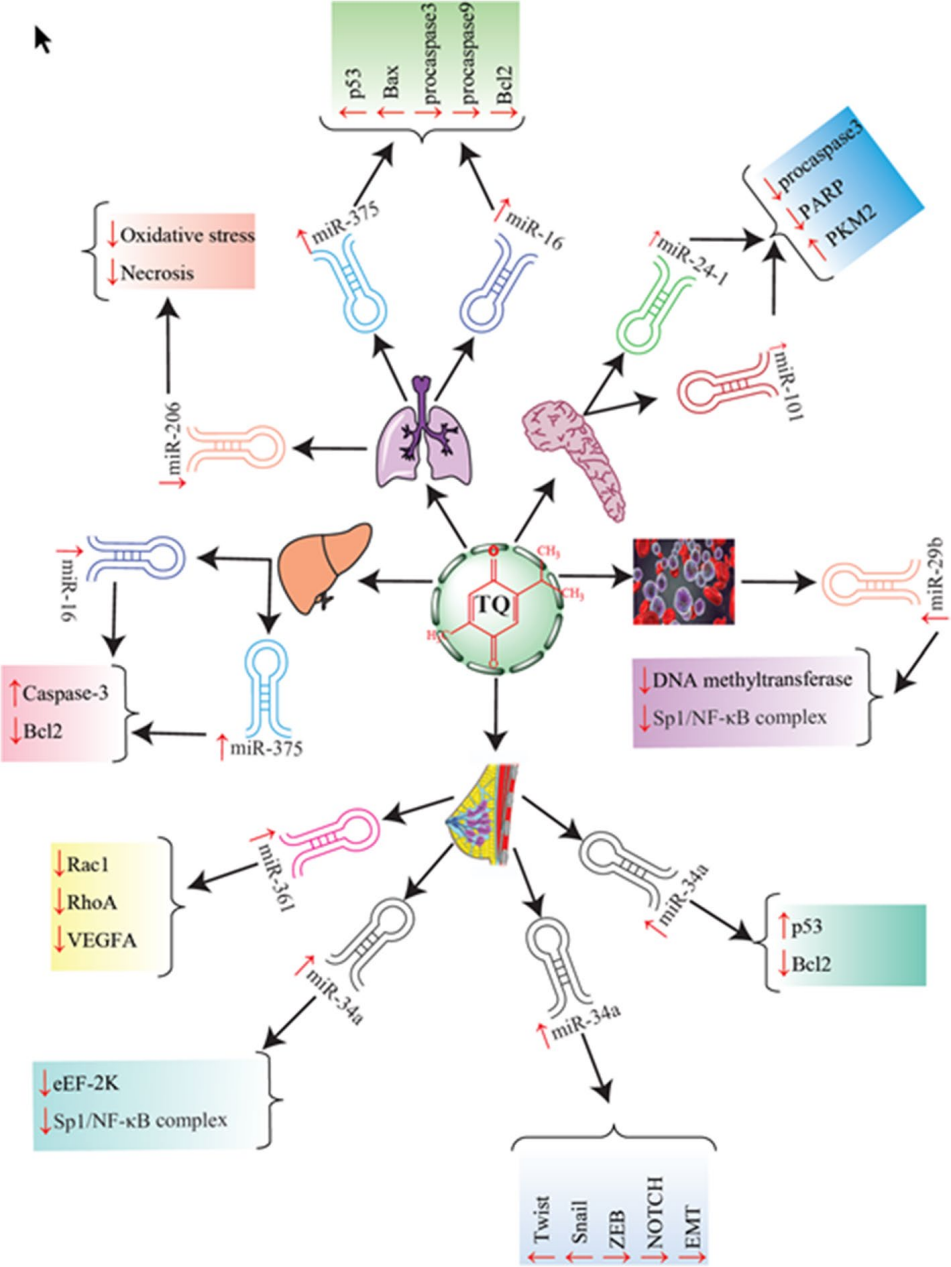
Graphic illustration of targeting various miRNAs with thymoquinone in cancer therapy

In spite of enhancement in conventional cancer therapeutic platforms, their clinical applications have not been significantly effective owing to disadvantages including low durability of primary cure, high possibility of recurrence, serious side effects through general toxicity as well as inadequate selectivity and also low life quality of suffering [5, 6]. Hence, exploration of modern remedial procedures for combating various cancer types along with the least possible adverse effects has received consideration in recent years. Nowadays, extensive research has been conducted into anticancer characteristics of plant bioactive compounds as revolutionary therapeutic agents thanks to their low toxicity, availability as well as affordable cost [7–10]. Accordingly, one of the promising natural pharmaceutics that has received a great deal of attention is thymoquinone. Thymoquinone (C_10_H_12_O_2_), chemically recognized as 2-isopropyl-5- methyl-1, 4-benzoquinone and with molecular weight of 164.204 g/mol (Fig. 1), is the prominent bioactive constituent of the volatile oil extracted from black seeds of *Nigella sativa* L [11]*.* Thymoquinone (TQ) exhibits various pharmacological attributes including antimicrobial, antioxidant, anti-inflammatory, antineoplastic, antidiabetic, antihypertensive, neuro- and cardio-protective effects. Therefore, TQ possesses capabilities to control enormous varieties of physiological disorders, particularly various cancer types [11–14]. Abundant in vivo and in vitro examinations have shown that TQ brings noteworthy anticancer and antineoplastic results against various cancer types such as breast cancer [15, 16], bone cancer [17, 18], pancreatic cancer [19, 20], lung cancer [21, 22] and liver cancer [23, 24]. Previous studies have demonstrated that TQ induces anticancer activity through affecting different biological pathways that are implicated in proliferation, apoptosis, angiogenesis, growth and metastasis of tumors [2]. However, low bioavailability, poor absorption and swift elimination in bile and urine are among the disadvantages by which TQ application as a therapeutic element is restricted [25, 26].

During the past decades, regulation of microRNA (miRNA) expression through TQ has been regarded as a novel strategy to combat cancers [27]. The purpose of this study was to review the trial examinations which have investigated the effect of the TQ/miRNA axis on signaling pathways interfering in cancer occurrence and progression. A brief summary of different investigations in the area of anticancer properties of TQ and different miRNAs is presented in Table 1 and Fig. 1. Also, several anticancer aspects of TQ/miRNA axis are introduced.

**Table 1:** Effect of thymoquinone (TQ) on the function of different miRNAs in the course of cancer therapy

Cancer type	Cell lines	Animal model	TQ dosage	miRNA type	Effect on miRNA	Mechanism of action of thymoquinone	References
Pancreatic cancer	PANC-1 MIA PaCa-2	–	In vitro: 6.25 μM	miR-24–1 miR-101	Up-regulation	Cleavage of procaspase3, PARP, increased PKM2 expression	[105]
Breast cancer	MDA-MB-231 MDA-MB-468	Mouse	In vivo: 3 μg/mL	miR-361	Up-regulation	Down-regulated Rac1, RhoA and VEGF-A, inhibit both metastasis and angiogenesis	[106]
Lung cancer	-	Mouse	In vivo: 10 mg/kg	miR-206	Down-regulation	Reduction of oxidative stress and necrosis formation, regeneration of the liver tissue	[110]
Breast cancer	MCF-7 cells	Mouse		miR-34a	Up-regulation	Up-regulation of p53, down-regulation of Rac1, Metastasis inhibition	[25]
Breast cancer	BT-549	–	In vitro: 5 μM	miR-34a	Up-regulation	Decreased levels of EMT-TFs including Twist and Snail, ZEB and NOTCH, control metastasis through down-regulation of EMT	[83]
Leukemia	MV4-11 and Kasumi-1	Mouse	In vitro: 10 μM In vivo: 15 mg/kg	miR-29b	Up-regulation	Dysfunction of DNA methyltransferases, dissociation of Sp1/NF-κB complex, induces apoptosis through activation of caspase-3 and caspase-8	[66]
Breast cancer	MDA-MB-231 MDA-MB-436	Mouse	In vitro: 15 μM In vivo: 100 mg/kg	miR-34a	Up-regulation	Suppressing NF-κB and eEF-2 K pathway	[65]
Liver cancer	HepG2, Huh7	–	In vitro: 10 μM	miR-16 and miR-375	Up-regulation	Up-regulated caspase-3, down-regulated Bcl-2	[114]
Lung cancer	A549	Mouse	In vitro: 5 μM In vivo: 5 mg/kg	miR-16 and miR-375	Up-regulation	Up-regulation of p53 and Bax, down-regulation of Bcl2, pro-caspase-3 and pro-caspase-9, induce apoptosis	[26]

## miRNAs: biogenesis and function

### Biogenesis

MiRNAs are considered as non-coding single-stranded regulatory RNAs (ssRNAs) with an average length of approximately 19–25 nucleotides generated by endogenous transcripts in a hairpin pattern [28]. The biogenesis of miRNA in humans is proceeded by a two-phase cleavage event occurring in both the nucleus and cytoplasm. Initially, at the nucleus stage, long primary miRNA transcripts (pri-miRNA) containing thousands of nucleotides are synthesized through RNA polymerase II [29, 30]. In fact, RNA polymerase II attaches to the promoters. Around half of miRNAs possess their own promoters and the other ones are present whether in introns or exons of genes, i.e. non-coding and coding regions of DNA [31]. The typical structure of each hairpin in pri-miRNA compromises a terminal loop, an upper stem, a lower stem as well as single-strand basal sections. The latter part is addressed by a 7-methylguanosine at the 5ʹ end and a polyadenylated tail at the 3ʹ end [32].

Subsequently, stem-loop structured pri-miRNAs are cleaved in a small hairpin shape containing 70–100 nucleotides and are labeled as precursor miRNA (pre-miRNA). This conversion is derived by the Drosha complex or Microprocessor including the nuclear endonuclease enzyme of RNase III or Drosha and its indispensable cofactor, diGerog syndrome critical region gene 8, as well as the double-stranded RNA (dsRNA)-binding protein [29]. The Microprocessor complex operates at about 11 base pairs above the ssRNA-dsRNA junction and splits the lower stem as well as the basal sections from the hairpin body. Thereafter, pre-miRNAs are transferred to cytoplasm via the protein Exportin 5 [28, 33, 34].

At the cytoplasmic stage, the Dicer (another type of Rnase III) and transactivation response RNA-binding protein (TRBP) complex acts on pre-miRNAs following its appearance in cytoplasm, separates the terminal loop and, as a result, small ds-miRNAs of 21–24 nucleotides in extent, known as small inferring miRNA (si-miRNA), are liberated. Thereupon, another TRBP engages Argonaute-2 (Ago-2) protein to load si-miRNA on the RNA induced silencing complex (RISC). Ago-2 singles out two strands of si-RNA based on their thermodynamic stability at the 5ʹ terminal. Consequently, the strand whose thermodynamic stability is lower, termed as the guide strand or the mature miRNA, leads to RISC through Ago-2, while the other one with higher thermodynamic stability is degraded by Ago-2 and is known as passenger mi-RNA [35–37].

### miRNA functions

MiRNAs play a crucial role in the process of RNA silencing through base–base interaction (base pairing) between miRNA and target mRNA. In this procedure, Ago proteins, as a decisive factor, have roles in various mechanisms such as translational repression, mRNA deadenylation and mRNA decay [29, 37]. Following formation of mi-RNA incorporated RISC or miRISC, it can be attached to the target sequence of mRNA, located in the 3ʹ untranslated region and followed by the coding sequence, and establish partial duplexes. Pairing interactions between miRISC and mRNA occur in the “miRNA seed” region. This region is recognized as nucleotide position 2 to 8 away from the 5ʹ end. It is proved although the remaining nucleotide sequence of 9 to 20 in the miRNA domain may participate in pairing interactions, a perfect complementarity between the miRNA sequences and the target mRNA is not required for the silencing phenomena [28, 31, 35, 36]. The narrow complementarity between miRNA and its action site is a massive advantage in the process of gene expression regulation and causes miRNAs to have the potential to prevent concurrently hundreds of various mRNAs from expressing [35, 38, 39]. Furthermore, various studies have revealed that over half of the protein-coding genes in the human body encompass more than one site for interacting with miRNAs and therefore can be controlled by miRNAs. Accordingly, it is not surprising that any dysregulation in the biogenesis and the function of miRNAs may be related to different human diseases which include various types of cancers, neurodegenerative as well as retinal and cardiovascular diseases [28, 31, 33, 37].

## Thymoquinone effects on miRNA regulation in signaling pathways

The term “epigenetic” is known as a branch of biological science considering the changes of genetic expression that are inheritable while involving no alteration in DNA sequences [40]. Various biological mechanisms may cause the expression of different genes to change and consequently modulate protein expression. DNA methylation/demethylation, histone acetylation/deacetylation and dysregulation of miRNA, circular RNAs and long non-coding RNAs are categorized as the main epigenetic systems regulating processes that directly manage the activity of different genes or proteins [41]. Accordingly, the slightest damage in the epigenetic structures results in dysfunction of proteins or genes and eventually different diseases including cancer [42]. Several investigations have indicated that dietary phytochemicals exhibiting anticancer characteristics are able to target disrupted cellular epigenetic systems and play crucial roles [43]. TQ is a well-known natural phytochemical compound that through targeting different miRNAs can suppress undesired epigenetic changes and has anticancer properties. For this purpose, TQ interferes in various signaling pathways and regulates expression of miRNAs in such a way that prevents cancerous cells from developing. In this section the mechanisms that cause TQ to act on miRNAs (whether oncogenic or tumor suppressor function) are introduced.

### p53 signaling pathway

P53, recognized as the “guardian of the genome”, is considered as one of the noted tumor suppressor genes that manages various cellular mechanisms, including apoptosis, cell cycle progression, cell death, cell proliferation inhibitors or even cell survival as well as different metabolic pathways [44]. The best way of describing the significant functions of p53 is to perceive its transcription character as either an activator or a repressor for expressing a great number of genes and miRNAs [45]. Various studies have demonstrated that p53 precisely causes the induction of miRNAs that act as mediators of tumor suppression processes such as encoding genes of the miR-15/16 family, miR-34, miR-107, miR-145, and miR-200 [46]. The mentioned miRNAs interfere in the process of tumor suppression and stress responses through arranging diverse fundamental processes including cell cycle development, cell survival, epithelial-to-mesenchymal transition (EMT), migration, differentiation and stemness [47]. Such properties arise about the explicit role of these miRNAs in the translation and protection of mRNAs that are the key elements of the forenamed processes. In the physiological stress conditions, particularly increased activated oncogene and also DNA damage, p53-regulated miRNAs are involved in various kinds of feedforward and feedback chains that ultimately lead to adequate cellular responses [48]. In consequence, the activity and expression of p53 are regulated by miRNAs as well, i.e. miRNAs not only regulate but also are regulated by p53 [49]. Therefore, the elaborate interplay between miRNAs and p53 should be regarded in the diagnostic and therapeutic management of numerous types of cancers. Up to this time, various inquiries have illustrated that the application of TQ as a therapeutic agent for various cancer types leads to prompt apoptosis and anti-proliferation impacts which are positively correlated with expression of p53 [20, 50, 51]. However, a few studies have examined the influence of TQ on miRNAs in the p53 signaling pathway.

Bhattacharya et al. (2015) encapsulated TQ molecules in polyethylene glycol-4000 nanoparticles and produced PEGylated-thymoquinone-nanoparticles (PEG-TQ-NPs) [25]. They investigated the theory that PEG-TQ-NPs might up-regulate p53, which afterwards would lead to expression of genes associated with miR-34a in breast cancer. Their results showed that the expression level of p53 was outstandingly elevated (in a time-dependent behavior) in human mammary carcinoma cell lines (MCF-7 cells) receiving PEG-TQ-NP treatment in a dosage of 5 μg/mL. Furthermore, a strong positive correlation was observed between the expression of p53 and the up-regulation of miR-34a in the MCF cells under treatment with PEG-TQ-NPs. They reported that, under the influence of PEG-TQ-NPs, the expression of p53 increased after 4 h while it occurred for miR-34a after 8 h, which showed that up-regulation of miR-34a is more controlled by p53 rather than PEG-TQ-NPs. To support this idea, firstly, MCF-7 cells were treated with pifithrin-a (p53 activity inhibitor) and then with PEG-TQ-NPs. The results obviously showed that in the presence of pifithrin-α, MCF-7 cells treated with PEG-TQ-NPs were not able to up-regulate the expression of miR-34a [25].

Upadhyay et al. investigated the impact of TQ nanoparticles (TQ-NPs) embedded in polyethylene glycol-poly (lactic-co-glycolic acid) and transferrin (TF, a kind of protein) on non-small cell lung carcinoma [26]. They reported that the treatment of A549 cells (human lung adenocarcinoma) with TF-TQ-NPs at the dosage of 5 μg/mL kg after 21 days led to an increased expression level of p53. Additionally, it was observed that the aforementioned treatment resulted in the incremented expression level of both miR-16 and miR-34a. They collectively demonstrated that TF-TQ-NPs successfully moderated up-regulation of p53, which in turn led to the activation of both miR-16 and miR-34a simultaneously [26].

### Nuclear factor-kappaB (NF-κB)

NF-κB is a class of acute phase proteins having the capability of transcribing various genes which are related to tumor suppressors, cytokine production and different cellular modulations including growth, proliferation, survival, apoptosis, angiogenesis and metastasis [52, 53]. In normal physiological circumstances, NF-κB is present in the cytoplasm and is inactive because the inhibitor of nuclear factor-kappaB (IκB) proteins cover the nuclear localization signals of the NF-κB proteins and prevent them from operating. However, in response to inflammatory mediators in tumor cells, IκB kinase causes IκB to phosphorylate and disassociate from NF-κB [54, 55]. Consequently, NF-κB is activated and translocated to the nucleus. At this point, NF-κB interferes in the transcription of genes controlling cell survival and thus the inappropriate regulation of NF-κB would be associated with uncontrolled cell growth and division and finally occurrence of various types of cancers [14, 56].

It has been demonstrated that various phytochemicals including TQ can act as an anticancer compound by suppressing the NF-κB signaling pathway [57–61]. Several mechanisms have been proposed for inducing anticancer properties of TQ through inactivation of NF-κB such as prevention of advanced glycation end product-induced NF-κB activation [61], inhibition of tumor necrosis factor-α (TNF-α)-induced NF-κB signaling [62], suppressive lipopolysaccharide-triggered NF-κB activation by inhibiting the transition of its p65 part to the nucleus [63] or deterrence of angiotensin II-promoter NF-κB activation and interleukin-6 (IL-6) expression [64]. Regarding miRNAs, Kabil et al. confirmed that TQ exerted anticancer effects by arresting NF-κB signaling in triple negative breast cancer (TNBC). In other words, TQ protects miR-603 in MDA-MB-231 and MDA-MB-436 by blocking the NF-κB pathway. It follows that miR-603 might exhibit anticancer characteristics [65]. In another study, it was ascertained that TQ (15 mg/kg of mouse body weight) through up-regulating miR-29b expression could obstruct the Specificity protein 1 (Sp1)- NF-κB feedback loop in mice bearing leukemia and eventually reduced the rate of tumor growth [66]. Furthermore, it was reported that in acute kidney injury, accompanied in 30% of cases by cancer, administration of TQ (20 mg/kg of body weight of rats) down-regulated the miR-34a expression by which the NF-κB expression was reduced. Recently, it was proved that miR-34a, by targeting the mRNA 3ʹUTR part of the nuclear factor erythroid 2-related factor 2 (Nrf2) gene, inhibits its expression. Thus, depletion of miR-34a may result in over-expressions and activation of the Nrf2/antioxidant response element (ARE) signaling pathway. Subsequently, Nrf2 causes NF-κB to degrade through reducing phosphorylation of IκB [67]. It should be clarified that Nrf2 is regarded as a redox-sensitive transcription signaling factor and is activated in response to oxidative or electrophilic incitements [68]. In redox-dependent conditions, Nrf2 is liberated, transferred to the nucleus, links to ARE and then plays a pivotal role in suppressing oxidative stress [69–71].

### Eukaryotic elongation factor 2 kinase (eEF-2 K) pathway

eEF-2 K is affiliated to the Ca^2+^/CaM-dependent α-kinase family. eEF-2 K can phosphorylate and consequently inactivate eEF-2 (at Thr56) and eventually prevent peptide chains from attaining enough elongation within mRNA translation [72, 73]. Recent investigations have shown that eEF-2 K is a significant signal transduction factor interfering in the most devastating cancers such as pancreatic, glioblastoma and breast cancers [74, 75]. There is some evidence on the relation between TQ and eEF-2 K in various cancer types. However, Kabil et al. (2017) suggested that, in TNBC, administration of TQ might inhibit tumor growth and progress by down-regulation of eEF-2 K signaling. In this targeting approach, TQ, by preventing NF-κB, activates miR-603, which in turn suppresses the eEF-2 K pathway [65].

### Phosphatidylinositol-4,5-bisphosphate 3-kinase (PI3K)/serine/threonine-specific protein kinase B (AKT) pathway

PI3K/AKT is a member of the kinase family [72]. It has been asserted that irregular overexpression of phosphorylated AKT is a distinctive attribute of different kinds of cancers [76]. AKT modulates numerous downstream target materials that control various cellular function including cell growth, proliferation, survival, glycogen metabolism and apoptosis [77]. Several observations have indicated that TQ, in the mechanism of anticancer action, induces the down-regulation of AKT and consequently breakdown of one of the endogenous deterrents of apoptosis, i.e. X-linked inhibitor of apoptosis proteins (XIAP) [53]. Low-expressed XIAP is associated with degradation of poly (ADP-ribose) polymerase (PARP) and activation of caspase proteins. In addition, inactivation or blocking of phosphorylation of AKT may cause the obstruction of B cell lymphoma 2 (Bcl-2) and ultimately induce apoptosis [72, 78]. In an investigation, it was revealed that receiving an injection of TQ (15 mg/kg of body weight) in leukemia bearing mice led to down-regulation of the PI3K/AKT signaling network via an Sp1-miR-29b negative feedback loop. In this approach, TQ by up-regulation of miR-29b could cause dysfunction of Sp1-NFκB and finally arrest the PI3K/AKT signaling pathway and induce apoptosis by activation of caspases [66]. In another study, it was suggested that TQ in a dose-dependent manner (0–15 μM of TQ) might indirectly regulate overexpression of the tumor suppressor miR-603 and finally dysregulate the PI3K/AKT signaling pathway in MDA-MB-231 and MDA-MB-436 cell lines of TNBC.

### Mitogen-activated protein kinase (MAPK)

The MAPK family has a crucial role in transmitting extracellular signals to intracellular objects. Similar to other signaling cascades, MAPK has regulatory roles in various organs and manages various biological processes such as cellular cycle, proliferation, migration and apoptosis [79]. It has been demonstrated that over-expression of MAPK is related to occurrence of numerous types of cancers [80]. Extracellular signal-regulated kinase (ERK), c-Jun N-terminal kinase (JNK) or stress-activated protein kinase (SAPK) and P38 MAPK are signal transduction pathways that belong to the MAPK family [81]. It has been indicated in several studies that TQ exerts its antitumor effect by means of induced apoptosis through triggering JNK and p38 MAPK signaling. Additionally, TQ by phosphorylation of JNK and ERK can induce apoptosis in cancerous cells [82]. Imani et al. (2017) reported that the EMT process could be suppressed in breast cancer cells by indirectly inactivation of MAPK in the MAPK/ EMT transcription factor (EMT-TFs)/TQ/miR-34a axis [83].

### Signal transducer and activator of transcription (STAT)

Previous investigations have revealed that STAT proteins manage various signal transducers including cytokines, hormones and growth factors and also play a principal function in tumor survival and proliferation [84, 85]. Both STAT3 and STAT5 as the chief members of STATs are cancer-causing proteins which arise from downstream mediators of the Janus kinase/signal transducer and activator of transcription (JAK)-STAT pathway [86]. In terms of different growth trigger signals, STAT 3/5 are phosphorylated through upstream kinases, in particular JAK, Src, KIT and FLT tyrosine kinases. Eventually, STAT 3/5 are dimerized and translocated to the nucleus [80, 87]. Although STAT activity is connected to the growth and progression of diverse cells and tissues, its over-expression has been incriminated in carcinogenesis [88]. Various studies have indicated that TQ can suppress the functions of STAT by different approaches such as inhibiting phosphorylation activity of JAK and Src kinases [89], preventing IL-6-induced AKT [90] as well as down-regulation of downstream targets of STATs, i.e., cyclin D1 and anti-apoptotic proteins Bcl-2 and Bcl-xL [89]. Concerning that STAT family signaling cascades are dormant transcription factors, a few experimental studies have directly investigated their behavior in the TQ-miRNAs axis. Pang and colleagues (2017) found that the suppression of Sp1-NF-κB as a result of the triggering effect of TQ on miR-29b led to inactivation of KIT and FLT tyrosine kinases, which are the principal regulators of leukemia. Conclusively, the inhibited tyrosine kinases activity caused dephosphorylation of STAT5 and Akt and finally the growth of leukemia tumors in lung and liver was arrested [66].

## Thymoquinone effects on miRNAs in cell progression and proliferation

As an extremely synchronized process, cell cycle progression is managed through a multitude of regulators and checkpoints in order to authenticate that the successive procedures of cell regeneration and proliferation are appropriately conducted [91]. In eukaryote cells, containing nuclei, cell division is separated into two main phases, namely the interphase, which includes Gap1, Synthesis as well as Gap2, and the cell division phase or mitosis [91]. In Gap1 or G1, the volume of cells increases, in the synthesis step, DNA replicates and in Gap2 or G2, the gap between DNA duplication and mitosis, cells continue growing. Finally in the mitosis stage or M, cells stop growing and instead, each cell splits into two identical daughter cells [92, 93]. Cell division and progression processes are modulated by cyclin-dependent kinases (CDKs) and their partner cyclins. Cyclins are categorized as regulatory proteins which arrange cell cycle processes by activation of CDKs. Both transition phases of G1 to S (G1/S) and G2 to M (G2/M) are regulated by CDKs [2, 11, 12]. It is corroborated that one of the generic specifications of malignant cancers that may cause cancer cell proliferation is abnormality in the cell cycle regulators [93]. Several studies have indicated that various anticancer components including TQ are capable of preventing cancerous cells from undergoing progression [94]. However, TQ has a unique strategy for combating each type of cancer. Paramasivam and co-workers (2016) reported that application of TQ in treatment of neuroblastoma cells could reduce the expression of proliferating cell nuclear antigen proteins in a dose-dependent fashion. Moreover, their findings indicated that TQ demonstrated the ability to reduce the expression levels of cyclin B1 (a component inducing cell transition from stage G2 to M) and CDK1 and, on the other hand, raise levels of expressed p53, p21 (cyclin dependent-kinase inhibitor—CDKI) and mRNA [95]. It should be noted that the interaction between cyclin B1 and CDK1 is a requisite for beginning M phase [96]. As a DNA clamp, PCNAs via protein–protein interactions play a prominent role in cell division for incorporation in different cell cycle regulation comprising replication, recombination and repair. Interaction of PCNA and CDKs is a paramount factor for development of all cell cycle stages, i.e. G1, S, G2 and M [97]. Various studies have demonstrated that TQ can arrest cell cycles by direct down-regulation of PCNA and cyclin B1 expression. Also, TQ by up-regulating p53, which in turn activates p21, can indirectly block CDK1. In fact, p53 through inducing p21 prevents cell cycle progression [12, 95, 97]. Gali-Muhtasib and colleagues (2004) observed that, at a dosage of 100 mM for 48 h, TQ blocked the cell cycle at G1 phase HCT116 colon cancer cell lines by the up-regulation of p21 and down-regulating of cyclin E [98]. Raghunandhakumar et al. (2013) observed anti-proliferative activities of TQ against hepatocellular carcinogenesis (HCC) at a concentration of 20 mg/kg. They noted that TQ triggered the up-regulation of p21 and down-regulation of cyclin D1, CDK4 and cyclin E and eventually obstructed cell progression through the G1 to S phase transition step [24].

Imran et al. found that TQ could disrupt the proliferation of mouse bearing spindle cancer cells by down-regulation of cyclin D1 as a result of p16 (as a CDKI) and p53 activity [13]. Parallel to the p53 signal pathway, TQ can prevent tumor cells from undergoing progression by targeting different signaling pathways such as ERK1/2 phosphorylation and eEF2K [11, 12]. Alongside the mentioned mechanisms, TQ exhibits knock-on effects on the progression and proliferation of cancerous cells by regulating miRNA expression [72]. As mentioned above, miRNA may function as either a tumor suppressor or an oncogene depending on regulated targets and pathways [99]. Recently, miR-603 has been recognized as a tumor suppressor in numerous cancer types which include glioblastoma, thyroid, breast, etc. [100]. A group of researchers found that TQ treatment (at 100 mg/kg of mouse body weight doses) of TNBC elevated the expressed miR-603, being enable to suppress expression of the eEF-2 K signaling pathway [65]. Various studies have demonstrated that miR-603 directly prevents eEF-2 k expression in TNBC [101, 102]. Additionally, it is proved in TNBC that eEF-2 K is exceedingly over-expressed whilst miR-603 is dramatically down-expressed. Thus, it is clear that insufficient expression of miR-603 may lead to TNBC progression and proliferation. eEF-2 K can manage the cellular cycle, particularly G1 to S transition, via modulating cyclin D1, PI3K/Akt, Src/Fak and the insulin-like growth factor receptor signaling axis [96, 103]. Therefore, miRNA by down-regulating eEF-2 K may inhibit TNBC tumors from growing and progressing [75, 104].

Pandita et al. reported that combining the influence of TQ and miR-101 and miR-24–2 at doses of 4.5 μM and 6.25 μM respectively might diminish proliferation of pancreatic cancer cell lines. This investigation indicated that TQ could act as an anti-proliferation agent against pancreatic cancer by up-grading both miRNAs which stop PARP and pyruvate kinase muscle isozyme 1 signaling pathways [105]. TQ encapsulated in hyaluronic acid nanoparticles (HA-TQ-NPs), at a concentration of 5 mg/kg of body weight of mouse suffering breast cancer, reduced the proliferation of cancerous cells through the up-regulation of miR-361, which suppressed regulation of Rac1, Ras homolog family member A (RhoA​) and the VEGF-A signaling axis [106]. It was thought for a long time that miR-206, whose homolog in mice is known as miR-206-3p, is an individual characteristic of skeletal muscles and it is vital for progression of this organ [107]. It was claimed miR-206 is exclusively expressed in skeletal muscles and its role in various functions such as myogenesis, hypertrophy, cardiac functions and growth of embryonic muscles is undeniable [108]. However, it was recently asserted miR-306 along with skeletal muscles is expressed in the pancreas, intestine, brain and liver [109]. In liver cancer, an investigation assayed the rate of expressed liver mRNA (miR-206) in an Ehrlich acid mouse solid tumor model (EAMST) following treatment with TQ (10 mg/kg of body weight) for four weeks [110]. Their results revealed high levels of up-regulated miR-206-3p and, as a consequence, oxidative stress and necrosis formation in EAMST models. Employing TQ as a treatment led to down-regulated miR-206-p3 and reduction of incidence of oxidative stress and necrosis [110].

MiR-34a, a member of the miR-34 family, was recently categorized as a tumor suppressor miRNA which is eliminated or down-regulated in a variety of cancer types including colon, pancreas, lung, liver and breast. Over-expression of miR-34a accompanied with inactivation of several oncogenic pathways has been identified [111, 112]. In breast cancer, Imani et al. reported that co-delivery of TQ (5 μM) and TmiR-34a in human metastatic breast cancers had a considerable potential to act as an anti-proliferative agent through suppressing TWIST1 and zinc finger E-box-binding homeobox protein (ZEB1) signaling in BT-549 cell lines [83]. Moreover, it was found in another study that TQ could exert its own anti-proliferative effects on breast cancer cells via significant up-regulation of miR-32a by which expression of Rac1 was diminished in both in vitro (1 μg/mL) and in vivo (5 mg/kg of body weight) approaches [25]. A group of researchers demonstrated that TQ in a dose-dependent fashion (0, 12.5, 25 and 50 μM) could extinguish the proliferation and activation of hepatic stellate cells (HSCs) by consecutive processes incorporating up-regulation of miR-30a, reduction of Snai1 values and suppression of epithelial EMT, resulting in inactivation of HSCs [113]. In renal acute injury, TQ (20 mg/kg of body weight of rats) triggered down-regulation of miR-34a that could prompt the expression of Nr2F and heme oxygenase-1, in turn inhibiting NF-κB signaling and, thereafter, preventing these cells from converting to cancerous ones [67]. In another study conducted by Pang et al. (2017), it was found that administration of TQ (15 mg/kg) into leukemia-induced mouse through up-regulations of miR-29b resulted in dissociation of the Sp1/NF-κB complex and finally termination of leukemia cell proliferation [66]. In HCC, TQ (10 μM) provoked apoptosis through up-regulating miR-16 and miR-375 and manifested its anti-proliferative aspect [114]. In lung cancer, the p53 signaling pathway operated as a linkage between TQ-NP-LF (5 μg/mL.kg) and both miR-16 and miR-34a. Under those circumstances, Bcl2 would be down-regulated and cell proliferation would be repressed [26].

## Thymoquinone effects on miRNAs in metastasis and angiogenesis

Both metastasis and angiogenesis processes play principal roles in cancer cell proliferation. Angiogenesis is described as a physiological process in which new blood vessels are established from preceding ones [12, 115]. This procedure is of considerable importance in the growth and progression of cells, wound healing and emergence of granulation tissues. However, it has been recognized as an indispensable stage in converting tumor cells from a benign phase to a malignant one. Angiogenesis is predominantly managed by chemical signals functioning as activators or inhibitors of tumor cell growth [116–118]. The concentration of chemicals incriminated in angiogenesis is an appropriate indicator for evaluating the degree of tumor aggression [119]. Cancer metastasis is another complicated process consisting of two chief phases: local invasion and remote migration [118, 120]. In this fashion, metastatic cells depart from primary tumors, enter blood vessels and finally attack adjacent tissues [121]. Recently, the restriction of angiogenesis and metastasis as a novel strategy for cancer therapy has attracted a great deal of attention. Beyond its suppressing roles against the proliferation and growth of cancerous cells, TQ has presented favorable features as an inhibitor of angiogenesis and metastasis processes [11, 13, 72, 81, 103]. Considering miRNAs, It has been reported that targeting TNBC with HA-TQ-NPs in both *ex ovo* and in vivo studies postponed cell migration and angiogenesis via the up-regulation of miR-361 and consequently inactivation of Rac1, RhoA and VEGF-A [106]. miR-361, located inside an intron which is between exon numbers 9 and 10 of CHM/choroideremia, has been detected in chromosome Xq21.2 and has major functions in various cancers [122].

Rac1 and RhoA are two noted constituents of the Rho GTPase family which regulate cell migration [123]. Rho-family small GTPases have been identified as influential chemical signals that manage different cellular qualities including protrusion, adhesion, morphology and thereby their locomotion [124]. Rac1 and RhoA have completely opposite functions and spatial locations. RhoA acts toward the cell rear and stimulates the cellular retraction all through the migration via actin-myosin filaments, while Rac1 functions at the leading edge of cells [125, 126]. Fascinatingly, RhoA and Rac1 conform in reciprocal inhibitory feedback cycles within protrusion-retraction loops. Appropriately, Rac1 has been revealed to decrease the activity of RhoA by numerous processes, particularly the downstream effector kinase PAK [127]. Conversely, it has been demonstrated that RhoA inactivates Rac1 via regulation of downstream effectors kinases ROCK1/2 [128]. Rac1 induces fabrication of dorsal stress fibers in the lamellar zone of cells by which the lamellipodium is formed [129]. Correspondingly, Rac1 is known as one of the most significant moderators of mesenchymal migration morphology (elongated spindle-shaped cells) in various cancers [130]. Furthermore, RhoA is one of the remarkable agents in formation of amoeboid migration morphology (rounded shaped without apparent polarity) [130]. Since Rac1 is involved in the mesenchymal morphology, it would be rational if RhoA stopped these steps. Equivalently, appearance of amoeboid morphology, as a consequence of high activity of RhoA, causes Rac1 to prevent RhoA from being activated [131]. Miscellaneous signaling molecules interfere in regulation of vasculogenesis and angiogenesis, including vascular endothelial growth factors (VEGFs), fibroblast growth factor (FGF), transforming growth factor-β and angiopoietin-1 and 2 [132]. VEGFs are homodimer glycoproteins that act as significant mediators of angiogenesis.

VEGFs bind to VEGF receptors (tyrosine kinase receptors) and eventually are expressed on vascular endothelial cells [133]. In the normal situation, VEGFs control vasculogenesis in embryonic evolution and are also required in wound healing, whereas VEGFs are up-regulated as a result of oncogenic influences [134, 135]. Tumor cells produce growth factors including VEGFs by which new abnormal vascular patterns throughout tumors are created that accelerate tumor growth [133, 134]. Thus, application of a number of factors that obstruct VEGF-A in order to halt the development and metastatic distribution of tumors has been propagated in recent years [136]. VEGF-A has been reported to induce TNBC cell migration through the autocrine signaling pathway on endothelial cells whilst paracrine signaling pathway causes VEGF-A to trigger angiogenesis progression [137, 138]. Bhattacharya et al. proved that the treatment of TNBC cell lines with HA-TQ-NPs significantly developed the expression of tumor suppressor miR-361 by which the expression of Rac1, RhoA and VEGF-A was down-regulated through blocking the 3ʹUTR part of their transcripts. Down-expression of both Rac1 and RhoA caused major disruption in the arrangement of actin stress fibers, which are one of the determining attributes of mesenchymal migratory morphology [106]. Moreover, down-regulated VEGF-A through preventing both autocrine and paracrine pathways may lead to inhibition of metastasis and angiogenesis respectively [139].

An investigation by Imani et al. showed that simultaneous implementation of pre-miR-34a and TQ as a novel therapeutic agent against metastatic breast cancer inhibited EMT signaling pathways by inactivation of TWIST1, Snail and ZEB1. They claimed that although miR-34a could alone inactivate EMT signaling, its combination with TQ caused its up-regulation and therefore their therapeutic capacity synergistically intensified. In this proposed approach, TQ not only suppressed hypoxia-inducible factor-1 alpha (HIF-1α), PI3/Akt and Wnt/β-catenin, known as inducers of EMT-TFs, but also up-regulated miR-34a, by which TWIST1, ZEB and Snail proteins were directly inactivated. As a consequence, migratory and invasive characteristics of cells could be discontinued [83]. EMT is considered as a complex biological process. As a result of this conversation, polarized epithelial cells lose their adherent properties and acquire new qualities including migration, invasion, resistance to apoptosis and extracellular matrix production [140, 141]. Convincing evidence has illustrated that several transcription agents, including Twist1,2, Snail, Slug and ZEB, which induce EMT processes, possess crucial roles in the metastasis of tumors and regulate through different signaling lines, namely Akt, STAT3, MAPK and Wnt [142].

Twist (Twist1,2) is a basic helix-loop-helix transcription factor classified as one of the significant EMT-TFs [143]. Various studies have reported that Twist (Twist1,2) acts as a pro-metastatic agent in various cancer including breast, prostate, bladder, hepatocellular carcinoma and so on [142–145]. Regulated Twist (Twist1,2) may result in tumor metastasis and invasion. In this way, expression of E-cadherin and N-cadherin, which are assay markers of EMT, is downregulated and upregulated, respectively, via Twist1 [146]. Zinc finger proteins are considered as the most typical components which bind DNA in eukaryotes. Snail, as a zinc finger transcription factor, blocks the promoter region of E-cadherin and suppresses the expression of cell adhesion proteins [147]. In this situation, firmly bonded epithelial cells are separated from each other and move into other sites. Consistently, expression of Snail is closely related to tumor metastasis thanks to its important role as a regulator of multitude signaling chemicals including epidermal growth factor (EGF), FGF, Wnt, Notch, TNF-α and cytokines [148]. The mentioned signaling pathways actuate Snail, which in turn by down-regulating E-cadherin and consequently cell movement, invasion and tumor development can prompt EMT [149].

Zinc finger E-box-binding homeobox protein (ZEB) is another member of the zinc finger family involved in regulating EMT within normal and pathological conditions [150]. The ZEB family consists of two main elements: ZEB1 and ZEB2. The ZEB family boosts the EMT process because of down-regulation of E-cadherin and up-regulation of numerous mesenchymal hallmarks by which cell migration, invasion and ultimately metastasis occur [151]. Similar to Twist (Twist1,2) and Snail, ZEB proteins are stimulated by disparate molecular signaling pathways such as HIF-1α, FGF, NF-κB, STAT3, Wnt and Notch [146]. Numerous examinations have revealed that disorganization of EMT-TFs in various cancer cell lines through activation of miRNAs is a promising approach to inhibit tumor progression and metastasis [152]. Established evidence has shown that miRNA-34a through the stem-loop structure could interact with the 3ʹ-UTR part of EMT-TFs and restructure the active binding sites of EMT-activation proteins. In this condition, expression of proteins related to EMT processes and post-transcriptional regulatory components are disrupted [83, 153].

In breast cancer cells, it was elucidated that employment of PEG-TQ-NPs could enhance the expression of miR-34a. In this circumstance, over-expressed miR-34a directly made the expression of Rac1 down-regulate by which actin was depolymerized. Finally, disruption of the actin cytoskeleton precipitated the establishment of lamellipodia and filopodia on the cell surface and consequently cell migration would decrease [25]. Irrefutable evidence Nietubyc [26].

## Thymoquinone effects on miRNAs in apoptosis and cell death

Apoptosis is a highly regulated process of programmed cell death which develops following pathological and physiological transitions and obliterates damaged, dead, aged and mutant cells [154]. As a matter of fact, apoptosis is a kind of pathway which purifies the biological structures from anomalous cells that may present a serious health threat against the body if it is not eliminated [2, 11, 13]. The intrinsic or mitochondrial pathway is one of the prime pathways which modulates apoptosis via the Bcl-2 (B-cell lymphoma-2) protein family. In this platform, permeability of mitochondrial membrane is promoted by different chemicals and, as a consequence, apoptosis-inducing agents are released [17]. Apoptogenic factors disrupt the membrane and function of mitochondria, which finally result in activation of several apoptogenic proteases such as caspases [155]. The other pathway for apoptosis is establishment of death receptors at the cellular surfaces that cause caspases to be activated [155, 156].

In most cancer variants, mutations bring about altering apoptosis regulating genes which include the Bcl-2 family, p53, caspases and PTEN. In contrast, under the effect of oncogenes, the expression of the MAPK family, consisting of extracellular signal-regulated kinase (ERKs), JNK/SAPK and the p38 group of protein kinase (p38 MAPK), NF-κB and interleukins, is elevated, leading to prevention of apoptosis [157, 158]. Accordingly, either dysregulation of apoptosis signaling systems or activation of anti-apoptotic compounds may lead to tumor growth and survival [50, 51, 63]. It is proved that a basis for cancer therapeutic approaches is encouraging apoptosis in order to eradicate malignant cells. Many studies have revealed that TQ can disrupt proliferation and induce apoptosis in cancerous cells but without any significant toxic effects on normal cells [23, 51, 63, 82, 89, 98]. Moreover, previous studies have shown that TQ can induce apoptosis in various types of cancer through regulating miRNAs as an activator or an inhibitor [26, 66, 105, 114]. In pancreatic cancer, the combination effect of TQ and gemcitabine (a chemotherapeutic agent, GCB) and miR-24–2 in PANC-1 pancreatic cancer cell lines was demonstrated. The results showed that the combination of TQ, GCB and miR-24 could promote apoptosis through down-regulation of Pro-caspase-3 [114]. Upadhyay et al. reported that targeting non-small cell lung carcinoma with TF-TQ-NPs led to upregulation of p53 that activated both miR-34a and miR-16. Then, the mentioned miRNAs by targeting Bcl2 could induce apoptosis in A549 cells. In addition, incorporation of TF-TQ-NPs into BALB/c mouse bearing A549 tumors showed that nanoparticles efficiently reduced the expression of Bcl2 by simultaneous activation of p53/miR-34a/miR-16 pathways [26].

In HCC, TQ induced apoptosis by up-regulating miR-16 and miR-375 expression [114]. Recently, various investigations have demonstrated that both miR-16 and miR-375 have a crucial role in controlling growth of tumors [159–161]. It has been shown that miR-16 may prompt apoptosis via suppressing Bcl-2 and NF-κB/MMP9 signaling. Also, it has been reported that miR-375 by targeting the gene of yes-associated protein (YAP) could alleviate its transcriptional functions [114]. YAP is a powerful oncogenic stimulus whose overexpression leads to HCC [162]. Insertion of TQ (at a concentration of 10 μM) into HepG2 and Huh7 cells caused the over-expression of both miR-16 and miR-375. Presence of these miRNAs up-regulated caspase-3 and down-regulated Bcl-2, which altogether encourage apoptosis in a synergistic manner and suppressed the growth of HCC cells [114]. In leukemia, TQ administration (15 mg/kg in mouse) into MV4-11 and Kasumi-1 led to expression of miR-29b, which in turns induced apoptosis through activation of caspase-3 and caspase-8 [66].

## Thymoquinone effects on miRNAs in DNA damage response

DNA damage is related to variations in physical or chemical features of DNA in a way that may affect genetic information including interpretation and transmission. Various exogenous and endogenous factors including free radicals, chemicals, and radiation are responsible for DNA damage [163]. The DNA damage response is the collective response activated as a consequence of DNA damage. The response involves identifying damage and checkpoint signaling and arrest, and ultimately leads to apoptosis and immune clearance [164, 165]. The major networks and signaling pathways by which DNA lesions are recognized and thereupon repaired include homologous recombination, non-homologous end joining, mismatch repair, nucleotide excision repair and base excision repair [164, 166]. Depending on the DNA damage, one (or more) of these signaling pathways may operate to safeguard the human genome. Genetic and epigenetic mutations may cause deficiency of gene and protein function and eventually cancer. The term epigenetics is defined as the variations in phenotypic traits that are heritable and do not cause any alteration in the DNA sequence [27, 167].

Manifold evidence has demonstrated that natural compounds such as TQ can directly and indirectly trigger and modulate epigenetic characteristics including histone acetylation or deacetylation and DNA methylation or demethylation [27]. In an in vivo investigation, Pang et al. found that TQ could dose-dependently (0, 3, 10 μM) induce DNA methylation in leukemia-bearing mice in the lung and liver by DNA methyltransferases. In this process TQ bound to catalytic sites of DNMT1, disrupted the Sp-1-miR29b loop, downregulated DNMT1 and hence DNA methylation was reduced. In this approach, TQ by inducing miR-29b, which in turn dissociates the Sp1-NF-κB complex, can down-regulate DNMT1 promotors and trigger apoptosis by the activation of caspases [66]. It should be noted that Sp1 is considered as one of the essential sequence-specific DNA binding proteins which manage various regulatory activities in the process of cellular transcription [168]. The anomalous expression and activation of Sp1 may induce the initiation and development of human cancers such as leukemia [66, 169]. Various observations have indicated that miR-29b specifically targets the sp1 gene and prevents it from being expressed. Accordingly, miR-29b, by inhibiting all cellular processes related to Sp1, can impede cancer development [170].

## Conclusion

Regardless of substantial progress in cancer treatment, the incidence of various cancers and frequency of cancer-associated deaths are still escalating. Because of insufficient efficiency and adverse side effects of conventional methods of cancer therapy, there has been a great deal of interest to apply phytochemicals as an anticancer agent. TQ as the chief natural component of *Nigella sativa* has been comprehensively employed in in vitro and in vivo investigations and a great variety of therapeutic attributes have emerged, which include anticancer aspects. It has been demonstrated that TQ has the capacity to prevent tumors from progressing via regulating miRNAs, which in turn manage signaling pathways involved in the pathogenesis of cancer cells such as proliferation, metastasis, angiogenesis, apoptosis and epigenetic machinery. P53, PCNA, cyclin D1, Bcl-2, NF-κB, TWIST (Twist1,2), ZEB, eEF-2 K, PI3K/Akt and Src/Fak are among the signaling pathways by which miRNAs moderate the anticancer effect of TQ. As outlined in this review, TQ can exert its multi-targeted anticancer properties more effectively by modulating miRNA expressions. However, there is a lack of adequate data about the bioavailability of the TQ/miRNAs axis and its roles in the DNA damage response, chemo-resistant cancers and also genetic and epigenetic machineries (Fig. 1). Thus, introducing the TQ/miRNAs axis as an anticancer platform is highly promising, but further investigation for enhancing its efficiency should be considered.

## Data Availability

Not applicable.

## Abbreviations

TQ: Thymoquinone
miRNAs: MicroRNAs
ssRNAs: Single-stranded regulatory RNAs
pri-miRNA: Primary miRNA transcripts
pre-miRNA: Precursor miRNA
TRBP: Transactivation response RNA-binding protein
si-miRNA: Small inferring miRNA
Ago-2: Argonaute-2
RISC: RNA induced silencing complex
EMT: Epithelial-to-mesenchymal transition
PEG-TQ-NPs: PEGylated-thymoquinone-nanoparticles
MCF-7 cells: Human mammary carcinoma cell lines
TQ-NP: TQ nanoparticle
TF: Transferrin
NF-κB: Nuclear factor-kappaB
IκB: Inhibitor of nuclear factor-kappaB
TNF-α: Tumor necrosis factor-α
IL-6: Interleukin-6
Sp1: Specificity protein 1
Nrf2: Nuclear factor erythroid 2-related factor 2
ARE: Antioxidant response element
eEF-2K: Eukaryotic elongation factor 2 kinase
PI3K: Phosphatidylinositol-4,5-bisphosphate 3-kinase
AKT: Serine/threonine-specific protein kinas B
XIAP: X-linked inhibitor of apoptosis proteins
PARP: Poly(ADP-ribose) polymerase
Bcl-2: B cell lymphoma 2
MAPK: Mitogen-activated protein kinase
ERK: Extracellular signal-regulated kinase
JNK: C-Jun N-terminal kinase
SAPK: Stress-activated protein kinase
STATs: Signal transducer and activator of transcriptions
JAK: Janus kinase
CDKs: Cyclin-dependent kinases
HCC: Hepatocellular carcinogenesis
HA-TQ-NPs: Hyaluronic acid nanoparticles
RhoA: Ras homolog family member A
EAMST: Ehrlich acid mouse solid tumor model
HSCs: Hepatic stellate cells
VEGFs: Vascular endothelial growth factors
FGF: Fibroblast growth factor
EMT-TFs: EMT transcription factors
EGF: Epidermal growth factor
ZEB: Zinc finger E-box-binding homeobox protein
HIF-1α: Hypoxia-inducible factor-1 alpha
YAP: Yes-associated protein
